# Asthma and COPD as etiologies of bronchiectasis: description and evolution.

**DOI:** 10.36416/1806-3756/e20250316

**Published:** 2026-03-05

**Authors:** Grace Oscullo, Jose Daniel Gomez-Olivas, Miguel Ángel Martinez-García

**Affiliations:** 1. Servicio de Neumología e Instituto de Investigación La Fe (IISLAFE), Hospital Universitario y Politécnico La Fe, Valencia, España.; 2. Centro de Investigación Biomédica en Red de Enfermedades Respiratorias, Instituto de Salud Carlos III, Madrid, España.

## TO THE EDITOR,

Bronchiectasis is defined as a usually irreversible dilation of the bronchial lumen,^1^ ^,^ ^2^ with associated thickening of the bronchial wall indicative of chronic inflammation, that is accompanied by characteristic symptoms such as chronic purulent cough and recurrent exacerbations.^1^ Chronic bronchial infection by pathogenic microorganisms, especially bacteria, is a common occurrence and is associated with disease progression.^3^

The condition has more than one hundred potential causes and related comorbidities,^4^ ^,^ ^5^ although in 30-50% of cases the etiology remains unknown, likely due to insufficient complementary testing. The most commonly known cause is a prior viral, bacterial, or mycobacterial infection (20-40%).^6^

From an epidemiological standpoint, bronchiectasis is the third most frequent chronic inflammatory airway disease after COPD and asthma. Both of these diseases have also been shown to cause bronchiectasis, especially in their advanced stages. Up to 50% of severe COPD cases^7^ and 25% of severe asthma cases may develop bronchiectasis as part of their natural history.^8^

The objective of this study was twofold: first, to assess the prevalence of COPD and asthma in the largest national and international bronchiectasis registries worldwide; and second, to analyze the temporal progression of this prevalence over a 20-year period.

In order to achieve this, information was obtained either through direct contact with the coordinators of the world’s largest bronchiectasis registries or, when this was not possible, through data published in the most recent scientific literature on the etiology of their bronchiectasis cases. Additionally, two Spanish registries were used for the temporal analysis of bronchiectasis prevalence: the historical bronchiectasis registry, launched in 2002 and closed in 2011,^9^ and the current registry, initiated in 2015 and active to this day.^10^

The percentages of COPD and asthma reported in the main registries range from 1.5% in China to 29% in the USA, and from 3.4% in Australia to 20% in the USA, respectively (Table 1). These striking discrepancies among registries regarding the proportion of patients with asthma or COPD have several possible explanations: (1) major differences in the diagnostic definitions used for asthma and COPD across countries; (2) variation in the underlying prevalence of COPD and asthma between countries; and (3) diagnostic errors that confound COPD/asthma with bronchiectasis, since these conditions often share clinical features such as smoking history and bronchial hyperreactivity. 

**Table 1:** COPD and asthma etiology in the largest available bronchiectasis registries in the world.

Etiology	EMBARC Europe	Historical Spain	RIBRON Spain	BRR USA	IBR India	ABR Australia	KMBARC S. Korea	China
Number	16,963	2,047	2,615	1,826	2,195	566	931	16,338
Period of inclusion	2015-2022	2002-2011	2015-ongoing	2008-2014	2015-2017	2016-2018	2018-2021	2020-2015
ETIOLOGY								
COPD	8.1%	7.7%	10.9%	20%	5.3%	3.4%	6.4%	4.9%
Asthma	6.9%	5.4%	7.8%	29%	2.5%	3.7%	5%	1.5%
Other	85%	86.9%	81.3%	51%	92.2%	92.9%	88.6%	93.6%

COPD, Chronic Obstructive Pulmonary Disease; EMBARC, The European Brochiectasis Registry; RIBRON, The Spanish Online Bronchiectasis Registry; BRR, Bronchiectasis and NTM Research Registry; IBR, Indian Bronchiectasis Registry; ABR, Australian Bronchiectasis Registry; KMBARC, Korean Multicenter Bronchiectasis Audit and Research Collaboration registry.

In any case, the highly disproportionate percentages of COPD (20%) and asthma (25%) reported in association with bronchiectasis in the USA are particularly noteworthy. This could be due to the distinctive nature of the US bronchiectasis registry^11^ in that a high percentage of its patients present bronchiectasis caused by non-tuberculous mycobacterial infection, as most patients are referred from specialized reference centers for such infections.

**Figure 1:**
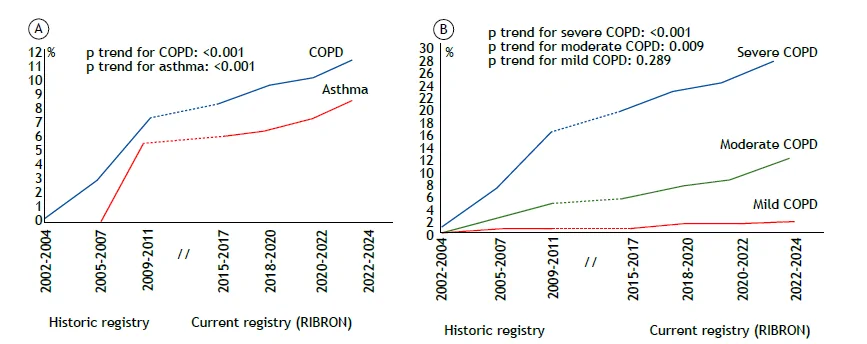
(A) Evolution of COPD and asthma as etiologies of bronchiectasis from 2022 to 2024. (B) Evolution during the same period depending on COPD severity. COPD, Chronic Obstructive Pulmonary Disease.

Regarding information related to the progression of COPD and asthma as causes of bronchiectasis in the registries, such data can only be extracted from the longitudinal information obtained from the historical and current bronchiectasis registries in Spain.^9^ ^,^ ^10^ Figure 1A shows that until 2005, asthma was not recorded as a cause of bronchiectasis, while COPD was first reported in 2002, accounting for 0.3% of all identified etiologies. However, since 2011, asthma and COPD have become significant causes of bronchiectasis, at 5.4% and 7.8%, respectively, and their prevalence has continued to increase-although more slowly-to the current percentages of 7.8% for asthma and 10.9% for COPD (p for trend <0.001), values that are similar to those observed in the European Registry.^12^

It is important to note that while there is evidence suggesting that COPD may cause bronchiectasis-given that some authors have reported patients with COPD who initially had no bronchiectasis but developed it after 7-8 years in the absence of any other identifiable cause^13^-in the case of asthma, this causal relationship has not yet been clearly established. Nevertheless, a significant correlation exists between asthma severity and the prevalence of bronchiectasis (20-25% in the most severe asthma cases).^8^

Figure 1B shows that when COPD is stratified by severity, using FEV_1_ in the presence of bronchial obstruction (FEV_1_/FVC < 0.7) as a marker (mild COPD: FEV_1_ > 80%; moderate COPD: FEV_1_ 50-79%; and severe COPD: FEV_1_ < 50%), the upward trend is similar across categories; however, the prevalence increases substantially in more severe forms, as expected, reaching 28% in the most severe category, compared to 11% and 1% in the moderate and mild categories, respectively. Numerous studies have shown that in COPD patients, the prevalence of bronchiectasis in severe forms of the disease ranges from 30-50%;^5^ the results of the present study are consistent with this evidence.

This progressive increase in both asthma and COPD over time may be explained by several factors. First, the growing use of high-resolution computed tomography in the diagnosis of bronchiectasis. Twenty years ago, high-resolution algorithms were not routinely applied unless specifically requested by the physician, and it is likely that not all bronchiectasis cases were identified using this method, which is now the diagnostic method of choice. Second, as the number of studies examining the relationship between asthma, COPD, and bronchiectasis has increased in recent years, healthcare professionals have become more aware of the association among these three conditions. Consequently, diagnoses of these overlap syndromes have also increased. 

Regarding the strengths of this study, it should be noted that it is the first to analyze, over a period of more than 20 years, the evolution of the diagnosis of asthma and COPD as etiological associations with bronchiectasis and to assess their increase over time. However, one limitation was the lack of data needed to categorize asthma severity. Moreover, in some cases, achieving a clear clinical distinction is not possible, and even after performing complementary tests, determining whether bronchiectasis is associated with asthma or COPD may remain challenging, since these conditions share many symptoms that can be indistinguishable. In the Spanish registries, to minimize this limitation, a detailed clinical history was consistently obtained along with the necessary complementary investigations. Furthermore, all patients were recruited from specialized bronchiectasis clinics, where bronchiectasis was the primary diagnosis.

In conclusion, the proportion of patients with asthma and COPD within the etiological spectrum of bronchiectasis has gradually increased over the past 20 years-initially rapidly and later more moderately-with consistently higher percentages observed for COPD. This trend carries important therapeutic implications.^14^ ^,^ ^15^ Nevertheless, studies are still needed to establish a true causal relationship between bronchiectasis and these two diseases, despite the clinical impression that such a relationship may exist. Moreover, caution is required when generalizing these results to other countries, given the wide geographical heterogeneity in the characteristics and etiologies of bronchiectasis. At present, therefore, we can only speak of an association between these conditions.
